# Sepsis: deriving biological meaning and clinical applications from high-dimensional data

**DOI:** 10.1186/s40635-021-00383-x

**Published:** 2021-05-07

**Authors:** Alex R. Schuurman, Tom D. Y. Reijnders, Robert F. J. Kullberg, Joe M. Butler, Tom van der Poll, W. Joost Wiersinga

**Affiliations:** 1 Center for Experimental and Molecular Medicine, Amsterdam UMC, Academic Medical Center, University of Amsterdam, Noord-Holland, Amsterdam, 1105 AZ The Netherlands; 2 Amsterdam Institute for Infection and Immunity, Amsterdam UMC, Noord-Holland, Amsterdam, 1105 AZ The Netherlands

**Keywords:** Sepsis, Multi-omics, High-dimensional data, Integration

## Abstract

The pathophysiology of sepsis is multi-facetted and highly complex. As sepsis is a leading cause of global mortality that still lacks targeted therapies, increased understanding of its pathogenesis is vital for improving clinical care and outcomes. An increasing number of investigations seeks to unravel the complexity of sepsis through high-dimensional data analysis, enabled by advances in -omics technologies. Here, we summarize progress in the following major -omics fields: genomics, epigenomics, transcriptomics, proteomics, lipidomics, and microbiomics. We describe what these fields can teach us about sepsis, and highlight current trends and future challenges. Finally, we focus on multi-omics integration, and discuss the challenges in deriving biological meaning and clinical applications from these types of data.

## Introduction

Sepsis was redefined in 2016 as life-threatening organ dysfunction caused by a dysregulated host response to infection [1]. This definition encompasses a group of patients that is heterogeneous in both clinical features and underlying pathophysiology. The pathophysiology of sepsis is multi-facetted and highly complex: it can involve concurrent immune overactivation and suppression, activation of the complement system, coagulopathy, endothelial dysfunction, gut microbiome disruption, and metabolic reprogramming of immune cells [2, 3]. While sepsis is a leading cause of global mortality, targeted therapies remain unavailable, making increased understanding of its pathophysiology crucial for improving clinical care and outcomes [4–6].

An increasing number of investigations is seeking to unravel the complexity of sepsis through high-dimensional data analysis. Technological advance increasingly enables -omics measurements of all constituents of a molecular layer—such as RNA, proteins, or metabolites—to provide an unbiased view of ongoing disease processes. Three common goals for utilizing this methodology in clinical sepsis research are studying the host response, developing diagnostics and discovering clinically relevant clusters. However, it remains challenging to combine these data into an overarching model of the mechanisms that govern sepsis pathophysiology and clinical outcomes.

Here, we summarize progress of high-dimensional investigations in the following major -omics fields, roughly following the central dogma of molecular biology: genomics, epigenomics, transcriptomics, proteomics, lipidomics, and microbiomics. Data are regarded as high-dimensional when the number of measured features exceeds the number of samples. We bring attention to studies that represent important advances in the field or exemplify a specific topic; this narrative review is not intended to constitute an exhaustive synopsis of the literature. We focus on what these fields can teach us about sepsis, and highlight current trends and future challenges (Table 1). High-dimensional investigations mainly using clinical data are not included, as we focus on research in human biological samples. Finally, in the last paragraphs we explore the potential and remaining challenges of integrating multiple -omics (multi-omics) data.

**Table 1:** Selected highlights of advances per -omics field

**Genomics**	**A number of genetic variants have been strongly linked to sepsis susceptibility and survival. The downstream effects of these variants are beginning to be uncovered**
Epigenomics	The epigenetic regulation of gene transcription is an emerging field of research in sepsis. First results show methylation of a large proportion of genes involved in the immunological response, which relates to clinical features like disease severity
Transcriptomics	Several diagnostic gene sets have been identified that can discriminate between types of inflammation. Transcriptome-based clustering can delineate pathophysiologically and prognostically relevant endotypes. In the near future, such tools could potentially guide personalized clinical therapy or the design of sepsis trials aimed at specific patient groups
Proteomics	Plasma proteomics revealed profiles related to clinical outcome, and perturbed energy metabolism pathways in patients with sepsis. Proteomics in specific cell subsets could pinpoint these alterations, possibly yielding targets for cell metabolism modulation
Lipidomics & metabolomics	Lipid- and metabolite signatures in plasma have been correlated with clinical outcomes in patients with sepsis. Cellular lipidomics and metabolomics could provide insight into structural changes and metabolic reprogramming of cells during infection
Microbiomics	Sepsis and antimicrobial therapy are associated with a disrupted gut microbiome, which has been linked to secondary infections and hospital readmissions. Next steps include identifying causal mechanisms and developing therapies aimed at restoring the healthy microbiome
Multi-omics	Simultaneously analyzing multiple molecular layers holds great potential for improving our understanding of sepsis pathophysiology. For inter-study comparability, transparency of the bioinformatic process must be a focal point

### Genomics

The field of genomics focuses on the structure, mapping, editing and function of genomes. One of the main goals of genomics is to identify genetic variants in the human genome that causally influence the risk of diseases. This can be accomplished by a genome wide association study (GWAS), in which millions of single-nucleotide polymorphisms (SNPs) throughout the genome are measured in a case–control design. Each subject is genotyped using a SNP array chip which directly measures usually up to 1 million variants across the genome, from which many more variants (e.g., > 90 million) can be imputed [7].

The fact that infectious diseases are known to have caused widespread mortality in children and young adults (before and during reproductive age) makes infectious pathogens arguably one of the strongest selective evolutionary forces to have acted on human populations [8]. Thus infectious pathogens are postulated to have shaped the human genome, such as by increasing the allele frequency of protective variants in immunity-related genes. The link between host genetics and survival from infectious diseases was strongly substantiated by the landmark study of Sørensen and colleagues, who reported that adult adoptees had a 5.8-fold increased risk of dying from infection if one of their biologic parents died of infection before the age of 50 [9]. Noteworthy is that this risk exceeded the risk of dying of cancer or cardiovascular disease. Discovering the genetic variants causally related to infectious disease mortality/survival, and understanding the corresponding physiological mechanisms present promising translational opportunities for novel therapeutics in sepsis.

To date, most GWA studies in the context of sepsis have focused on outcomes after developing sepsis, such as 28-day mortality [10, 11]. In this type of study design, all cases and controls have sepsis and the case/control status is defined by the patient’s mortality outcome. These studies have revealed SNPs in genes such as FER, but the exact mechanisms through which these polymorphisms exert their protective or harmful effect remains to be elucidated. For instance, the presence of the s4957796 SNP in the FER gene was found to significantly improve survival in sepsis patients, possibly through the role of FER in the regulation of cell adhesion, leukocyte recruitment and intestinal barrier dysfunction [10]. It is important to note that the *FER* variant was only found to be associated with survival in sepsis due to pneumonia and was not associated with mortality in a more heterogeneous cohort of patients with sepsis due to either abdominal infections or pneumonia, indicating that different mechanisms may be involved depending on the site of infection [11].

In addition to identifying variants that influence survival after developing sepsis, a second goal is to find variants that influence the risk of developing sepsis. A meta-analysis of candidate gene studies (targeting suspected genes) reported a number of SNPs in genes coding for pattern recognition receptors and cytokines significantly associated with the risk of developing sepsis, including TLR4rs4986790 and TNFArs1800629, respectively, both investigated in more than 25 studies [12]. As Toll-like receptors (TLRs) are vital for innate immune cells to recognize pathogens, and tumor necrosis factor alpha (TNF-α) is an important pro-inflammatory cytokine, genetic variants of these genes could potentially strongly influence the host response during infection. To date, it remain uncertain whether such SNPs indeed mediate the risk of developing sepsis. GWA studies with a design in which cases are sepsis patients and controls are from the general population could further investigate this, although to our knowledge such studies have not yet been conducted.

### Epigenomics

One of the main ways in which gene transcription is regulated is through epigenetic changes such as DNA methylation and histone modification; for instance, DNA methylation at a gene promoter region generally acts to repress gene transcription [13]. When studied on an omics scale, this approach is known as epigenomics. In the context of sepsis, recent studies are beginning to unravel how epigenetics plays a role in sepsis pathogenesis. Binnie and colleagues performed an epigenome-wide DNA methylation analysis of whole blood samples from 68 septic and 66 non-septic critically ill adults [14]. They discovered 668 differentially methylated regions (DMRs) of which the majority (61%) were hypermethylated. Next, enrichment analysis of the DMR-containing genes was performed, showing that pathways related to an anti-inflammatory and T-helper 1 type immune response were enriched with hypermethylated genes. Conversely, pathways including negative regulation of IFNγ production were enriched with hypomethylated genes. Finally, the authors found certain sets of methylated genes that were correlated to the need for vasopressors and disease severity, suggesting a link to clinical features. Another study measured global DNA methylation specifically of monocytes in patients with sepsis [15]. Sepsis was associated with changes in methylation of genes relevant to the function of monocytes, including those involved in inflammation mediated by chemokine–cytokine signaling (hypermethylated) and MHC class II protein complex (hypomethylated), supporting the role that DNA methylation plays in regulating gene expression in sepsis.

A further mechanism known to regulate the transcriptome, although not categorized under epigenomics, is through non-coding RNAs such as long non-coding RNAs and micro RNAs. This has recently been studied in sepsis using a next-generation micro array, performed on leukocytes. It was found that long non-coding RNA and, to a lesser extent, small non-coding RNA were significantly altered in sepsis relative to health [16]. Future mechanistic studies on sepsis may aim to integrate epigenomics and transcriptomics to determine how much of the variance in transcriptomics is influenced by methylation, histone modification and non-coding transcripts.

### Transcriptomics

A wide variety of RNA molecules, transcribed from the genome, exert diverse functions in the production of proteins and the regulation of gene expression. Most transcriptomic studies in sepsis examined messenger RNA (mRNA)—and thus the expression of genes that may lead to the production of the corresponding protein—but this focus has expanded to regulatory non-coding forms such as microRNA and long non-coding RNA, as we noted in the previous paragraph. When measuring the transcriptome, microarrays allow for the detection of a large number of predefined genes, whereas next-generation RNA-sequencing detects all RNA present, including novel and alternatively spliced transcripts [17].

The vast amounts of data generated through transcriptomics have facilitated the discovery of novel diagnostics for sepsis. While sequencing all RNA in a biological sample is costly and time consuming, combinations of genes with validated diagnostic value can be rapidly and relatively inexpensively assessed via partially automated qPCR methods. For instance, several gene sets derived from whole blood leukocytes have been validated in multiple cohorts to distinguish systemic inflammation due to infection from ‘sterile’ systemic inflammation without an overt infectious cause [18]. Such gene sets include the Sepsis MetaScore [19], SeptiCyte™ LAB [20], and the *FAIM3:PLAC8* ratio [21]. These diagnostics, perhaps in sequence with other tests to improve their overall predictive value, could theoretically aid in reducing unnecessary antibiotic exposure in the ICU—to avoid harmful side effects and antimicrobial resistance—or, at the very least, prompt physicians to consider alternative non-infectious diagnoses that may require different treatments.

Transcriptomic data has also been used to cluster patients with sepsis into subgroups that may not immediately be apparent at the bedside, but do share clinically relevant pathophysiological characteristics—so-called endotypes. If these endotypes are robust and rapidly identifiable, they could allow for a precision medicine approach to sepsis: treatment decisions could be based on specific underlying biological processes rather than the relatively aspecific clinical definition of ‘suspected infection with organ failure’ [18]. In recent years, several endotypes have been characterized and validated: endotypes A and B in pediatric sepsis [22]; sepsis response signature (SRS) 1 and 2 [23]; Mars 1 through 4 [24]; and, using all publicly available transcriptome data at the time (including those used in the preceding three studies), the inflammopathic, adaptive and coagulopathic endotypes [25]. At a molecular and pathophysiological level, some of these endotypes appear to capture overlapping phenomena. For instance, the SRS2, Mars3 and adaptive endotypes largely describe the same group of patients with an upregulation in adaptive immunity genes (associated with lower mortality) [24, 25]. These endotypes could theoretically be used predictive enrichment (identifying patients with certain pathophysiological characteristics more likely to respond to certain treatments), but this will first require answering the many open questions on topics such as stability of endotypes over time, generalizability, and implementation [18, 26]. Studies that translate these endotypes into clinical practice remain scarce, but differential responses to treatment have been described: in a post-hoc analysis of the VANISH trial, hydrocortisone administration was associated with higher mortality in the non-immunosuppressed SRS2 endotype [27], whereas in pediatric sepsis endotype B, characterized by higher glucocorticoid receptor signaling, corticosteroid treatment was linked to favorable clinical outcomes [28].

Assessing gene expression in whole blood leukocytes, or specific cell fractions, can be used to study the septic host response in an untargeted manner. For instance, Claushuis et al. stratified critically ill patients with sepsis based on platelet counts and found that—even when matched for severity of disease and other confounding factors—the most severely thrombocytopenic patients exhibited a more disturbed host response (e.g., overexpression of genes related to the complement system) when compared with patients with normal platelet counts [29]. Using publicly available data, Zador et al*.* demonstrated substantial overlap in the gene expression pathways involved in the response to pulmonary sepsis, abdominal sepsis, and trauma [30]. By utilizing CIBERSORT, a method to derive leukocyte population fractions from bulk RNA data [31], they found higher mortality rates in patient groups that were characterized by lower abundance of circulating neutrophils.

An emerging and promising technique in the field of sepsis transcriptomics is single-cell RNA-sequencing, in which cells are separated into individual droplets and combined with unique RNA-barcoded beads that allow for subsequent identification of the transcriptome of each cell [32]. These techniques generate vast amounts of data within each subject, but the high financial cost still preclude the larger sample sizes obtained in bulk transcriptomic studies. This limits both power for between-subject comparisons and the generalizability of results. An advantage of single-cell RNA-sequencing is that it enables researchers to identify novel cell types and states that are lost in bulk data: Reyes et al.recently performed single-cell RNA-sequencing in peripheral blood mononuclear cells of a cohort of 29 septic patients (primarily with urinary tract infections) and found a novel monocyte state (named “MS1”) that was virtually absent in healthy controls and patients with urinary tract infection but without sepsis [33]. Importantly, the authors managed to assess the robustness of this MS1 state in several ways: they validated the MS1 transcriptomic signature in external bulk RNA-seq data, they generated cells with MS1-like characteristics by stimulating bone marrow precursor cells with lipopolysaccharide (LPS), and they defined surface markers that allowed cell sorting of MS1 cells [33]. Another study that investigated seven patients with sepsis and four healthy controls confirmed previously reported alterations in sepsis at the single cell level, such as downregulated genes related to HLA-DR and alterations in energy metabolism pathways in the monocyte clusters [34]. A third study that included seven septic patients—three of whom also developed acute respiratory distress syndrome (ARDS)—reported a clear upregulation of genes related to type I interferon signaling in ARDS, potentially driven by a virtual absence of *SOCS3* (a negative regulator of cytokine signaling) in all monocyte clusters in these patients [35].

### Three main applications of high-dimensional data in sepsis

1. Study the host response to elucidate key mechanisms of sepsis pathophysiology, potentially guiding future therapies.
2. Develop diagnostics to improve bed-side testing and personalized medical treatment.
3. Uncover clinically relevant clusters within the heterogeneous group of sepsis patients.

### Proteomics

Proteomics is often seen as the next step down the -omics hierarchy, following genomics and transcriptomics. It entails the analysis of proteins that are produced or modified by an organism, covering composition, activity and structure. Measurements are often performed through gel electrophoresis, antibody immunoassays or liquid chromatography followed by mass spectrometry [36].

Proteins consist of amino acids, which have been assembled in ribosomes based on the translation of a mature mRNA blueprint. This mature mRNA is derived from precursor RNA—the primary transcription of DNA—which has been modified by for instance 3′ polyadenylation, 5′ capping and intron splicing to produce a functional mRNA molecule. After ribosomal translation, proteins are often altered by the addition or removal of specific molecules (such as phosphate or methyl groups), which can markedly alter protein function. Taken together, these post-transcriptional and post-translational modifications render the proteome highly dynamic and obscure its direct relation to the genome and transcriptome.

As the final product of this network of transcription, translation and modification, proteins can provide valuable mechanistic insight and/or serve as biomarkers. Several research groups have performed untargeted proteomics analyses in sepsis (the use of single targeted proteins as biomarkers has been reviewed elsewhere [37, 38]). For instance, De Coux et al. compared survivors and non-survivors of sepsis in a small cohort of patients presenting to the emergency room, and found more than 90 plasma proteins that were exclusively present in one of the two groups [39]. Using the Kyoto Encyclopedia of Genes and Genomes database the authors determined specifically upregulated pathways in surviving patients, such as the extrinsic coagulation and complement cascades. Another investigation reported age-related proteomic changes in a population of 19 elderly septic patients [40]. In a more extensive, multi-step effort, Langley et al. sought to characterize both the plasma metabolome and proteome in plasma of patients with sepsis upon their presentation at the emergency department and 24 h later [41]. The study design entailed three sets of patients with sepsis or systemic inflammatory response syndrome (SIRS): a discovery cohort of 150 patients, a validation set of 52 patients and a second validation set of 90 patients. Through cross-correlation and hierarchical clustering of the proteome and metabolome the authors recapitulated known metabolic reactions, unveiling analytes and pathways—such as a profound defect in fatty acid beta-oxidation—that differentiated between survivors and non-survivors. Interestingly, in this study the plasma metabolome and proteome could not differentiate sepsis from severe sepsis nor septic shock in survivors. Alterations in lipid metabolism pathways were also reported in another study, comparing plasma of 23 healthy controls, 20 sepsis survivors and 13 sepsis non-survivors at hospital admission and 7 days later [42]. Downregulation of apolipoproteins and alterations in cholesterol metabolism delineated sepsis patients from healthy controls, while dysregulation of the actin cytoskeleton pathway was more pronounced in sepsis non-survivors than in survivors.

Although these investigations highlight the potential of proteomic data, it also underlines the limitation of plasma measurements. It is challenging to draw mechanistic conclusions from diverging signatures in specimens such as plasma or urine [43], as the origin of the analytes often remains uncertain. This issue can be sidestepped by focusing on a more specific sample, such as platelets or neutrophils [44, 45], or resolved by validating findings in vitro.

### Lipidomics and metabolomics

Lipids are a prerequisite for the existence of cells. Lipids have both important structural and bioactive functions. By forming a lipid bilayer they are the essential building blocks of all membranes, providing structure and compartmentalization. As bioactive molecules, lipids play a key role in many cellular processes such as cellular energy metabolism, transport of mediators and cell–cell signaling [46]. In this context, the study of metabolites (metabolomics) is closely related to lipidomics as it is often measured with the same methods, mainly through chromatographic separation followed by mass spectrometry identification.

Tens of thousands different lipid species exist in the human body, which can be divided into eight main classes and many more subclasses based on chemical structure and properties [47]. Due to the variability of lipids, the range of biological concentrations and limitations of previous detection tools, our grasp of the lipidome is trailing that of the genome and transcriptome.

In sepsis, most studies investigating lipids thus far have focused on the potential role of specific lipids as a plasma biomarker. For instance, high-density lipoprotein cholesterol (HDL-C) levels decrease during sepsis, which has been associated with worse clinical outcomes [48]. HDL-C can bind and isolate potentially harmful lipids derived from pathogens, and has, therefore, been hypothesized to play a protective role in bacterial infections [49]. A more untargeted approach has been employed by Mecatti et al., who measured a part of the plasma lipidome in 21 patients with SIRS and 21 patients with sepsis [50]. Multiple lipid species, such as glycerosphingolipids and prostaglandins, were more abundant in the sepsis group, while l-octanoylcarnitine was found to be most relevant for prognostic classification, discriminating between survivors and non-survivors. Another study associated lipid signatures with therapy responsiveness 21 patients with septic shock, showing that lysophosphatidylcholine levels only increased in treatment responsive patients during the disease course [51].

‘Eicosanoids’ are arguably the most extensively studied lipids in the context of infections. Roughly divided into pro-inflammatory mediators and pro-resolving mediators, these bioactive lipids are enzymatically produced from poly-unsaturated fatty acids in leukocytes [52]. Non-steroidal anti-inflammatory drugs have been used to inhibit pro-inflammatory eicosanoids (such as prostaglandin) for decades. However, it has only recently been recognized that eicosanoids can also actively mediate the resolution of inflammation. In a murine sepsis model, it has been shown that intervention with pro-resolving mediators such as resolvins and protectins increased phagocytic uptake and bacterial clearance, lowering antibiotic requirements [53]. Eicosanoids may also serve as biomarkers: a study by Dalli et al*.* reported that levels of lipid mediators in plasma of critically ill patients with sepsis correlated with mortality and the development of ARDS [54].

Lipids and metabolites have a wide array of cellular functions. As such, measuring the metabolome and lipidome of isolated immune cells during infection could be highly informative of alterations in key cellular processes. For example, an increasing number of studies show that changes in energy metabolism pathways, also called metabolic reprogramming, can alter immune cell functionality [55]. To illustrate, it was recently shown that macrophage phenotypes—ranging from pro-inflammatory to anti-inflammatory—can be shaped by controlling fatty acid oxidation [56]. Khalic et al. showed in metabolic signatures in serum of 33 critically ill patients that both an increase and a decrease in mitochondrial fatty acid beta-oxidation products was related to mortality, hypothesizing that a “corridor of safety” might exist, consisting of a certain range in which cellular fatty acid metabolism must be maintained [57].

Understanding the role of lipids and metabolites may not only provide valuable insights in the inner workings and structural changes of immune cells, but may also elucidate host–pathogen interactions [58, 59], and pave the way for future interventions. Collectively, current studies indicate that lipidome and metabolome alterations may play an important role in the host response during sepsis, although the field remains in an explorative phase.

### Microbiomics

The human microbiome comprises trillions of microbes that colonize the human body, primarily bacteria in the gut [60]. The development of culture-independent technologies—such as 16S rRNA and shotgun metagenomic sequencing—has facilitated the investigation of these microbial communities and their role in health and disease. Numerous experimental and epidemiological studies have demonstrated an important, yet incompletely understood role of the gut microbiome in sepsis [61]. Hospitalization for reasons known to cause a disrupted microbiome, such as infections and antibiotic treatment, increases the risk for readmission to the hospital with sepsis [62]. Several prospective cohort studies have examined microbiome changes in critically ill patients and showed that these patients had a reduction of obligate anaerobes and overgrowth of potentially pathogenic bacteria (such as *Staphylococcus* and *Pseudomonas* spp.) [63–66]. It should be noted that these findings may be confounded by the antibiotic treatment patients receive, as well as by the other interventions such as enteral/parenteral feeding and the use of gastric-acid inhibition and sedatives [61].

Disruptions of the microbiome may not be limited to the gut, as it has been shown that the lung microbiome is altered during critical illness and can become enriched with gut-associated bacteria [67]. In a murine model of sepsis, lung bacteria were most likely to originate from the lower gastrointestinal tract, while in humans with ARDS the level of gut-specific bacteria in broncho-alveolar fluid was abundant and associated to disease severity [68]. Furthermore, a recent proof-of-concept study showed an association between decreased lung microbiome diversity and increased mortality in patients with extrapulmonary sepsis [69].

The methods by which the microbiome is measured continue to evolve. The amplification and sequencing of marker genes is a commonly used and cost-effective method to obtain an overview of one type microbial community (e.g., 16S rRNA for bacteria), but seems insufficient to meet the challenges of this field. For a more complete picture, researchers could take into account all communities of microorganisms that may be present in a certain sample and integrate the bacteriome, virome, fungiome and protozome [70]. Metagenomic sequencing measures all genes in a microbiome sample—including viral and eukaryotic DNA—and thereby enables higher taxonomic resolution and inference of functional capacity [71]. Current metagenomic practices use short-read sequencing to simultaneously sequence the mixture of microbial genomes, but the results are sub-optimal due to the fact that short-reads can align to the genomes of multiple microbial genomes [72]. Long-read sequencing has been used to mitigate this problem, and could facilitate a more robust measurement of all communities.

Whereas the composition of the microbiome can be determined in great detail, current interventions (such as probiotics or selective decontamination) are often aspecific and utilize a one-size-fits-all approach. Advancing our understanding of the microbiome in health and the dysbiosis associated with sepsis may aid in the development of personalized microbiome-based therapies [73].

### Integrating multi-omics data

**Fig. 1:**
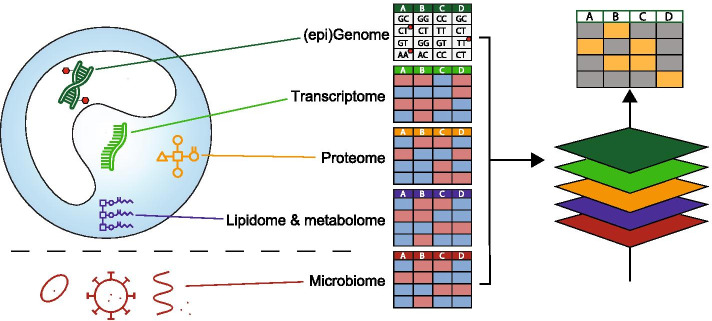
Schematic overview of different molecular layers measured by -omics technologies. Integrating these layers through multi-omics analysis can yield a more holistic view of biological processes and uncover novel connections between layers

In the words of the essayist and poet Jorge Luis Borges: “Everything touches everything”. All molecular layers discussed in the previous chapters are interdependent and influence each other continuously (Fig. 1). Integration of these layers of molecules can yield a more holistic view of biological processes and uncover novel connections between layers. Advances in bioinformatics methods have enabled the integrated and concurrent analysis of two or more -omics layers (multi-omics), but only few studies in the field of sepsis have attempted this (the various computational tools available for multi-omics analysis are reviewed elsewhere [74, 75]). The first study to integrate genomics and transcriptomics in sepsis was performed by Davenport and colleagues [23], who proposed that “individual heterogeneity in the transcriptomic response to sepsis might be modulated by genetic variation”. They studied this using expression quantitative trait loci (eQTL)—these are genomic loci (SNPs) for which the genotype is significantly associated with gene expression. In their sepsis cohort the genes at cis-acting eQTLs (where the SNP and the gene locally coincide on the chromosome) were found to be enriched in pathways relevant to sepsis such as viral respiratory infection and cellular immune response. The cis-eQTL genes included *TLR4* and *TNF* establishing that in sepsis patients, leukocyte gene expression of key immune regulators is indeed influenced by their genetics.

Multi-omics may also improve diagnostics by increasing the dimensionality of the data: Wong et al*.* developed a classifier combining transcriptomic markers and serum proteins that discriminated between survivors and non-survivors in children with septic shock, and outperformed an earlier classifier based on serum proteins alone [76]. Combing genomics with measurements of metabolites and cytokines revealed the methionine salvage pathway as regulator of sepsis that can predict prognosis in patients [77].]. As mentioned earlier, Langley et al*.*, could differentiate survivors and not-survivors in sepsis using an integrated proteomics and metabolomics approach [41].

Multi-omics methods are still in their infancy and beset by several challenges. First, many multi-omics tools require normalization of the data, noise filtering and other preprocessing steps. Without standardized approaches for this, it becomes difficult to compare studies and generalize results. Second, each ‘omic’ operates on a different timescale, making it challenging for single time point measurements to detect causal effects between layers. Third, there is no consensus at this time on how to choose the right analysis technique for a particular combination of -omics, perhaps with the exception of eQTLs. Subramanian and colleagues suggested to classify tools by technique (such as Bayesian, network-based methods or factorization) and by the biological question of interest they are able to address (such as defining subgroups, discovery of biomarkers or obtaining insights into pathophysiology) [75]. Such consensus-based recommendations may be helpful for researchers to choose the analytical method that best fits their data and research question.

## Discussion

Reductionism—the belief that every single process in nature can be broken down in and explained by its constituent parts—is invaluable in grasping complexity. We know that cells are not two-dimensional, that pathways are not linked by arrows, and that many reactions only occur after reaching some critical (in)balance, often with non-linear consequences. Still, testing and visualizing processes this way help us to dissect very complex problems such as sepsis. All science is reductionist to some extent, but -omics technologies allow for a more all-encompassing representation of the processes under investigation. These types of studies are open to findings outside the scope of our current understanding. Nevertheless, after all high-dimensional data integration and analyses, we still rely on results derived from traditional experimental research for the interpretation and validation of findings.

Despite the increasingly sophisticated analysis methods, measuring one substrate at a single moment is unlikely to capture a biological process in its entirety. Perturbations in sepsis are not necessarily confined to specific compartments, such as the broncho-alveolar space or peripheral blood, but more likely part of system-wide and interconnected changes. We know that processes change over time and as sepsis progresses, which leads to temporal heterogeneity. Thus, studies would ideally include longitudinal measurements of multiple biological compartments. Importantly, even the most sophisticated measurements and bioinformatics analyses cannot compensate for poorly collected samples or biased study designs.

Certain -omics fields, like transcriptomics, have adopted the mandatory practice of sharing raw data. This has already led to prolific re-use of data generated in one or multiple cohorts, and will allow for the meta-analysis of multiple datasets. Meta-analyses can provide increased power and efficiency for discovering and consolidating data patterns with potential clinical relevance, such as different sepsis endotypes. While more data reduces random error, it does not prevent bias, and the risk of bias is especially high when one is unaware of the intricacies of the datasets and the confounders therein. Meta-analyses of high-dimensional data would be wise to adopt the methodological rigor employed by traditional meta-analyses in clinical epidemiology, such as those by the Cochrane collaboration, and to collaborate with the researchers that published the dataset for individual patient level data.

## Conclusions

Tremendous progress has been made in sepsis research, but several overarching challenges must still be overcome. Multi-omics approaches, combining molecular layers and biological compartments, have the potential to improve our understanding of sepsis pathophysiology, help develop more rapid diagnostics, and facilitate personalized medical management.

## Data Availability

Not applicable.
